# Dietary Acid Load and Bone Health: A Systematic Review and Meta-Analysis of Observational Studies

**DOI:** 10.3389/fnut.2022.869132

**Published:** 2022-05-06

**Authors:** Fatemeh Gholami, Sina Naghshi, Mahsa Samadi, Niloufar Rasaei, Khadijeh Mirzaei

**Affiliations:** Department of Community Nutrition, School of Nutritional Sciences and Dietetics, Tehran University of Medical Sciences (TUMS), Tehran, Iran

**Keywords:** dietary acid load, fractures, bone mineral density, meta-analysis, bone

## Abstract

Findings on the association between dietary acid load (DAL) and bone health are conflicting. This study aimed to summarize available studies on the association between DAL and risk of fractures or bone mineral density (BMD) in adults. Online databases including PubMed, Scopus, and Embase were searched for relevant studies published up to June 2021, using pertinent keywords. We identified observational studies (cohort, case-control, and cross-sectional) investigating the association between DAL and risk of fractures or BMD, then selected studies following these reported criteria: RRs with corresponding 95% CIs for the relationship between DAL and fracture risk; correlation coefficients for the association between DAL and BMD; and mean ± SD of BMD values across the categories of DAL. Overall, 17 studies with 80545 individuals were included. There was no significant relationship between the PRAL and fracture risk (Pooled RR: 1.18; 95% confidence interval 0.98 to 1.41, *I*^2^ = 60.6%). Moreover, a similar association was observed between the NEAP and fracture risk (Pooled RR: 1.41, 95% CI: 0.79 to 2.52, *I*^2^ = 54.1%). The results of five studies from four publications revealed no significant association between dietary PRAL score and femoral and spinal BMD (WMD femoral = −0.01, 95% confidence interval: −0.02 to 0.01, *I*^2^ = 76.5%; WMD spinal = −0.01, 95% CI: −0.03 to 0.01, *I*^2^ = 56.7%). However, being in the highest category of NEAP was significantly associated with a lower femoral and spinal BMD (WMD femoral = −0.01, 95% CI: −0.02 to −0.00, *I*^2^ = 82.1%; WMD spinal = −0.02, 95% CI: −0.03 to −0.01, *I*^2^ = 93%). It was showed that adopting diets high in acidity was not associated with risk of fractures. We also found a significant negative relationship between NEAP and BMD. However, DAL based on PRAL was not associated with BMD.

## Introduction

Osteoporosis is a major health threat characterized by skeletal fragility and microarchitectural deterioration (1–3). Factors associated with bone health include sex, physical inactivity, alcohol consumption, smoking, loss of estrogen, and diet (4). Previous studies have documented that adequate calcium intake and appropriate nutrient status are essential for the prevention of osteoporosis and its devastating consequences. In addition to individual dietary components for improving musculoskeletal health, a balanced diet is also important (5). The high dietary acid load (DAL) represents a diet rich in nutrients that are metabolized to non-carbonic acids such as sulfuric acid in amounts greater than the alkaline bicarbonate produced by the combustion of organic salts like potassium chloride provided by fruit and vegetable consumption (6). Therefore, foods containing sulfate and phosphorus such as meat, fish, cheese, grain products, and rice are considered acidogenic (7, 8). While foods rich in bicarbonate, potassium, magnesium, and calcium, such as fruit, legumes, vegetables, red wine, and potatoes, are considered alkaline (9). Epidemiological and scientific researchers have indicated that following a Western dietary pattern, composed largely of acidogenic foods, might lead to excess endogenous acid production, and consequently, low-grade metabolic acidosis (6–9). Metabolic acidosis releases calcium from the bone matrix mediated for increasing osteoclastic resorption to maintain homeostasis, weakening the bone and making it susceptible to fracturing (9, 10). Such evidence raises the question of whether long-term adherence to diets with high acidity potential might contribute to the loss of bone mass and osteoporosis (11–13). Findings on the association between dietary acid load (DAL) and bone health are controversial. While some studies have documented that a high DAL was associated with a reduction in the density and structure of bone mass (14–18), others failed to find such evidence (19, 20). This inconsistency has also been documented for observational studies that investigated the association of DAL with fracture risk (14, 21). A recent meta-analysis did not provide evidence for a protective effect of alkaline diet on bone health (22). However, several studies have been published since the release of that meta-analysis. Moreover, that meta-analysis did not consider (NEAP) and potential renal acid load (PRAL) separately. Therefore, in the current study we aimed to summarize the available evidence on the association between DAL and risk of fractures and bone mineral density (BMD) in adults, by considering two major methods for estimating DAL including PRAL and NEAP.

## Methods

This meta-analysis was conducted using the preferred reporting items for systematic reviews and meta-analyses (PRISMA) guidelines.

### Search Strategy

We developed and performed the literature search (FG), and two reviewers (FG and MS) screened the titles and abstracts. The same two reviewers independently assessed the full text of the articles for eligibility. Differences were resolved by discussion. PubMed, Scopus, and Embase databases were searched to identify relevant papers published up to June 2021. We used a search strategy that included truncated free text and exploded MeSH terms (Supplementary Table 1). No restrictions were imposed on language of publications. We identified additional studies by manually searching the reference lists of retrieved articles.

### Inclusion Criteria

Published studies were included if they had the following criteria: (1) observational studies (cohort, case-control, and cross-sectional) conducted in adults, (2) reported RRs or HRs with corresponding 95% CIs for the association of DAL and fracture risk or correlation coefficients for the association between DAL and BMD or mean ± SD of BMD values across categories of DAL.

### Exclusion Criteria

We excluded letters, comments, reviews, meta-analyses, and ecological studies.

### Data Extraction

Required data were extracted from qualified studies by two independent investigators (MS and SN) using an abstraction form: first author's name, publication year, study design, demographic characteristics of participants (age range or mean age and sex), geographical location, sample size, number of fracture cases, method of assessing outcome, dietary assessment tool, RRs for fracture, mean ± SD of BMD values in the highest and lowest categories of DAL or regression coefficients for BMD and confounding variables that were adjusted in the statistical analyses. Numerical estimates were extracted from graphs by using a web plot digitizer (23). Any disagreements were also resolved by discussion and consensus.

### Quality Assessment

The methodological quality of studies was evaluated using the Newcastle–Ottawa scale (NOS), designed for nonrandomized studies (24). Studies with six or more points were considered as high-quality papers.

### Statistical Methods

We included the RRs and 95% CIs for fracture to compare the highest vs. the lowest categories of DAL through the meta-analysis. In the analysis of continuous data (BMD in g/cm^2^) the means and corresponding SDs in the highest and the lowest categories of DAL were used to calculate the weighted mean difference (WMD) as the effect size in the meta-analysis. In cases of the absence of SD data, we calculated it by using SE and sample size.

To calculate the overall effect size, a random-effects model was used to take between-study heterogeneity into account. Cochran's Q test and the *I*^2^ statistic were used to assess heterogeneity between studies. For the *I*^2^ statistic, we considered the *I*^2^ values of 25, 50, and 75% as low, moderate, and high between-study heterogeneity, respectively (25). When studies reported risk estimates stratified by sex or other variables, we first pooled the subgroup estimates by using fixed-effects meta-analysis. Then, the obtained pooled risk estimate was included in the main meta-analysis. Subgroup analyses were conducted based on some important variables to detect possible sources of heterogeneity. Overall effect size in each subgroup was calculated by a random-effects model. If ≥10 studies were available, we explored the possibility of publication bias by inspecting funnel plots and conducting Egger's test (26). We removed each study one after another to perform sensitivity analysis and examine the influence of each study on the overall estimate.

## Results

### Literature Search

**Figure 1:**
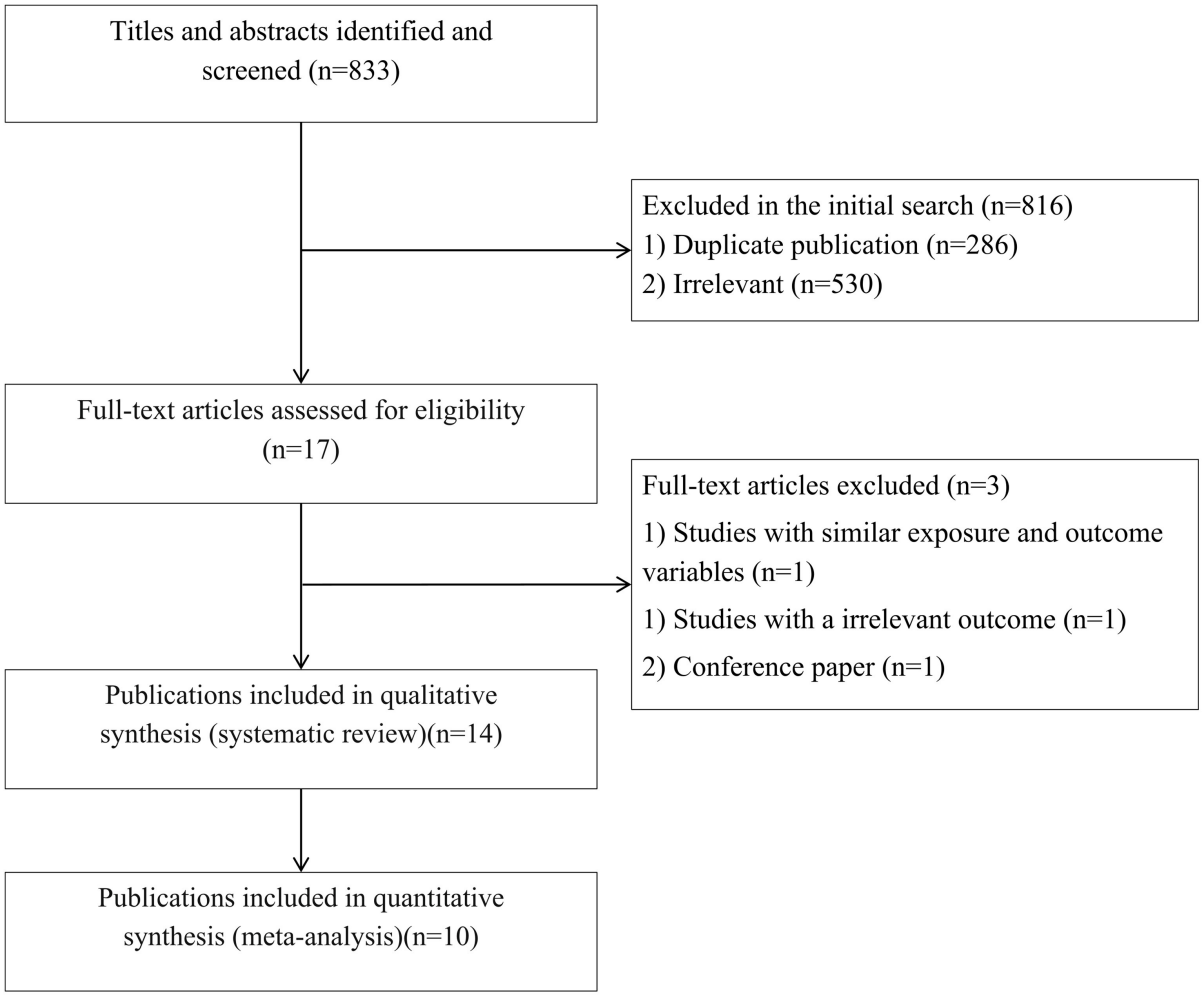
Flow diagram of study selection.

In total, we identified 833 articles in our initial search. After excluding duplicate papers (*n* = 286) and unrelated studies (*n* = 530), 17 relevant studies remained for full-text review (Figure 1). Among these, three publications were excluded due to same exposure and outcome variables (14), irrelevant outcomes (27), and being a conference paper (28). Finally, 14 publications were included in the systematic review (15, 17, 19–21, 29–37) and ten publications were included in the meta-analysis (17, 19, 21, 29–31, 33–36).

### Characteristics of the Included Studies

Supplementary Table 2 shows the characteristics of studies included in the current systematic review and meta-analysis. The number of participants in these studies ranged from 161 to 36,217 in the 29-92 age range. Of the 14 included articles with 80,545 participants, seven publications enrolled only women (15, 29–33, 35). Among the remaining articles, three of them reported results in both sexes, separately (17, 34, 36). In total, three included papers carried out in the United States (16, 31, 35), and 11 publications performed in non-US countries (15, 19–21, 29–31, 33, 35–37). To evaluate DAL six publications applied food frequency questionnaires (21, 29, 30, 33–35) and four articles used food recall or record (17, 19, 31, 32, 36, 37). Four studies reported beta for the association of DAL and BMD. Because these studies presented different units for BMD, including T-score, Z-score, and g/cm^2^ that their conversion to each other was not possible, we only included these studies in the systematic review. Because their conversion to each other was not possible (19, 31, 32, 37). Moreover, two studies investigated the association of DAL and bone ultrasound measures [broadband ultrasound attenuation (BUA)] and reported BMD values based on dB/MHz; therefore, we included them in the systematic review (15, 36). All included studies except four (15, 30, 32, 33) were of high quality based on the NOS.

### Findings From Meta-Analysis on Fracture Risk

#### PRAL and Fracture Risk

**Figure 2:**
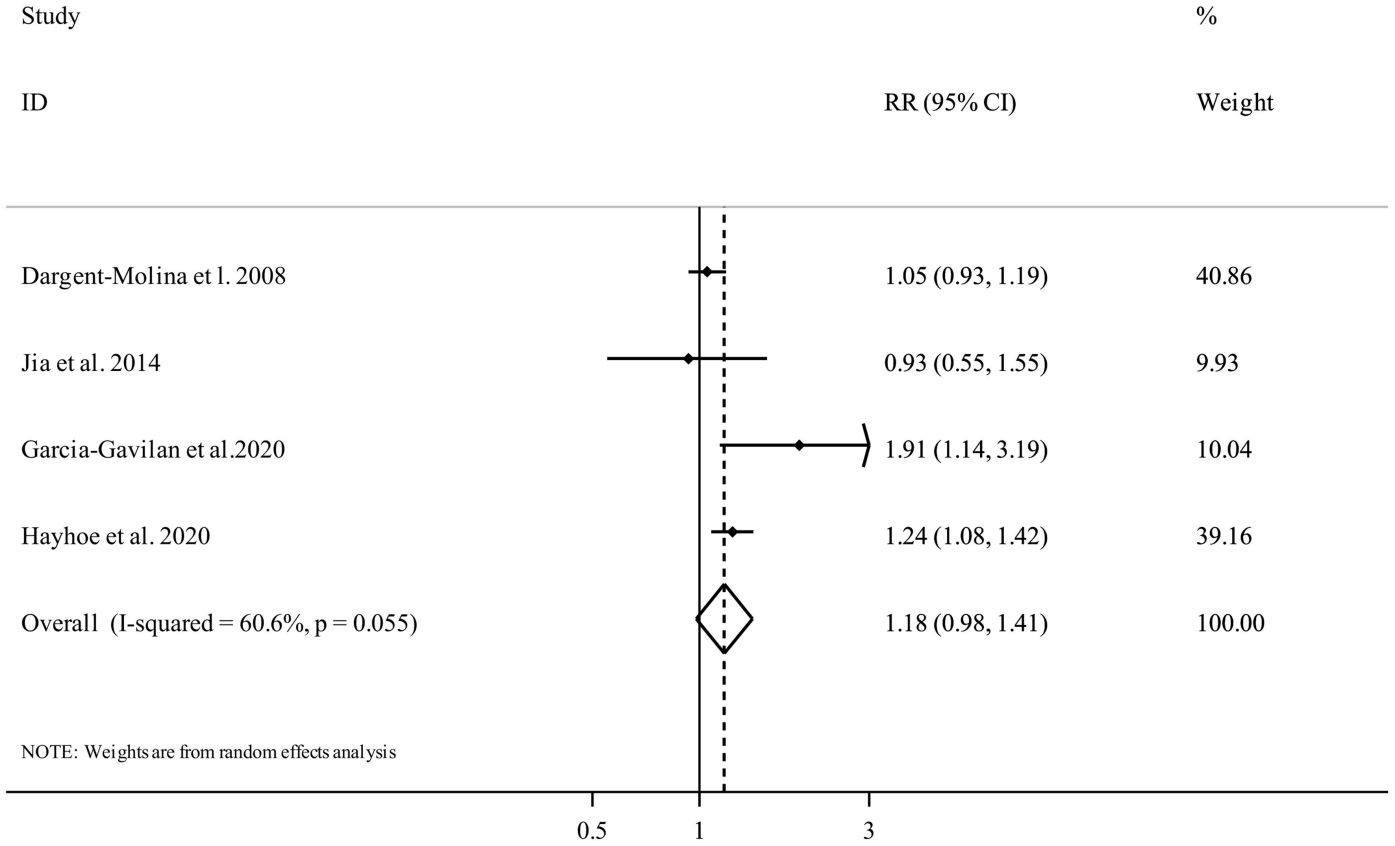
Forest plot for the association between PRAL and fracture risk in adults aged >18 years expressed as the comparison between the highest and lowest categories of PRAL. Horizontal lines represent 95% CIs. Diamonds represent the pooled estimates from the random-effects analysis. RR, relative risk; CI, confidence interval.

Four publications (19, 21, 31, 36) included a total of 63,386 participants and 4,846 fracture cases examined the association between PRAL and fracture risk. The summary RR for fracture risk, comparing the highest and the lowest categories of PRAL, was 1.18 (95% confidence interval 0.98 to 1.41, *I*^2^ = 60.6%, P_heterogeneity_ = 0.05) (Figure 2), indicating a non-significant association.

#### NEAP and Fracture Risk

**Figure 3:**
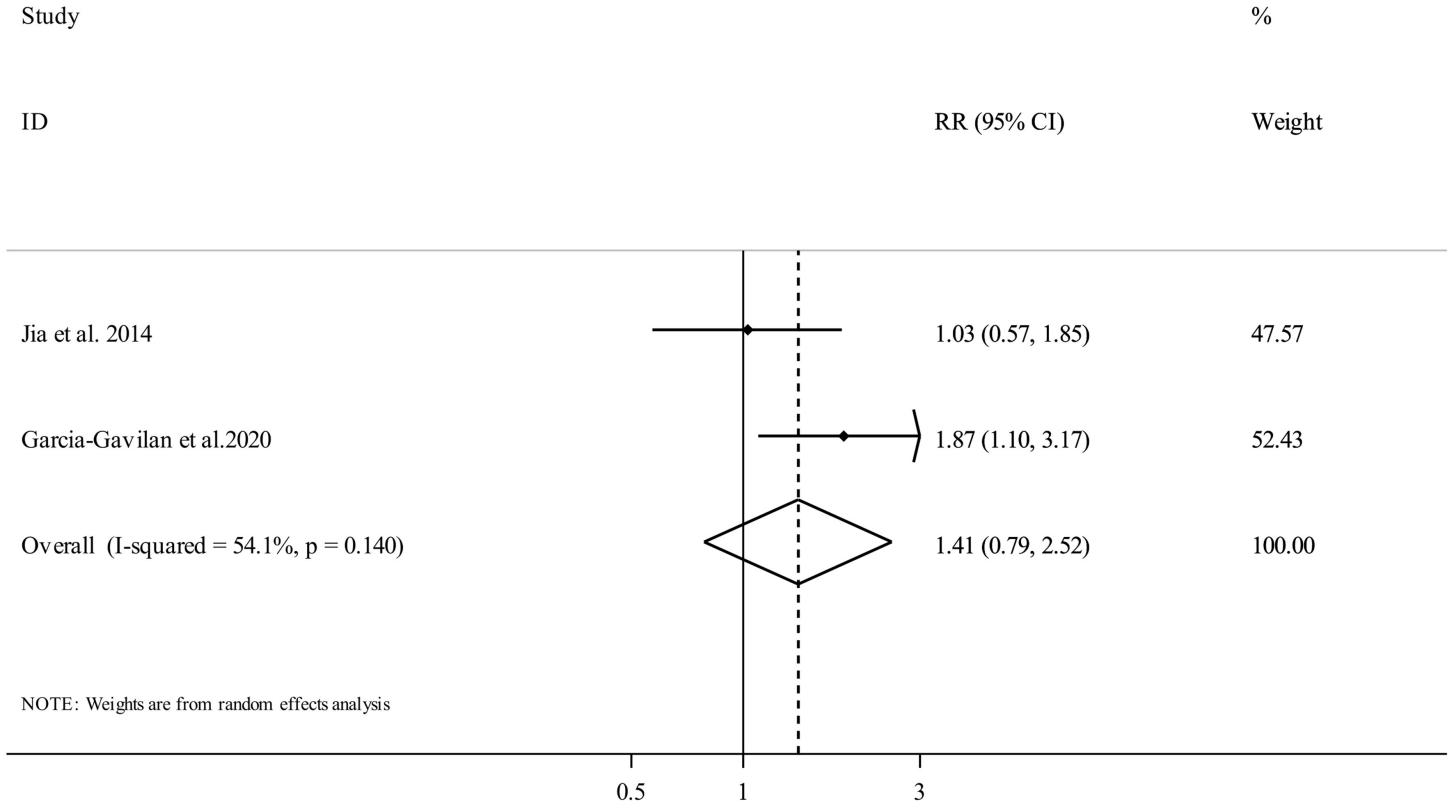
Forest plot for the association between NEAP and fracture risk in adults aged >18 years expressed as the comparison between the highest and lowest categories of NEAP. Horizontal lines represent 95% CIs. Diamonds represent the pooled estimates from the random-effects analysis. RR, relative risk; CI, confidence interval.

Two publications (19, 21) with 1,731 participants and 245 fracture cases, evaluated the association between NEAP and fracture risk. Comparing the highest vs. the lowest categories of NEAP demonstrated no clear significant association (Pooled RR: 1.41, 95% CI: 0.79 to 2.52, *I*^2^ = 54.1%, P_heterogeneity_ = 0.14) (Figure 3).

### Findings From Meta-Analysis on BMD

#### PRAL and BMD

**Figure 4:**
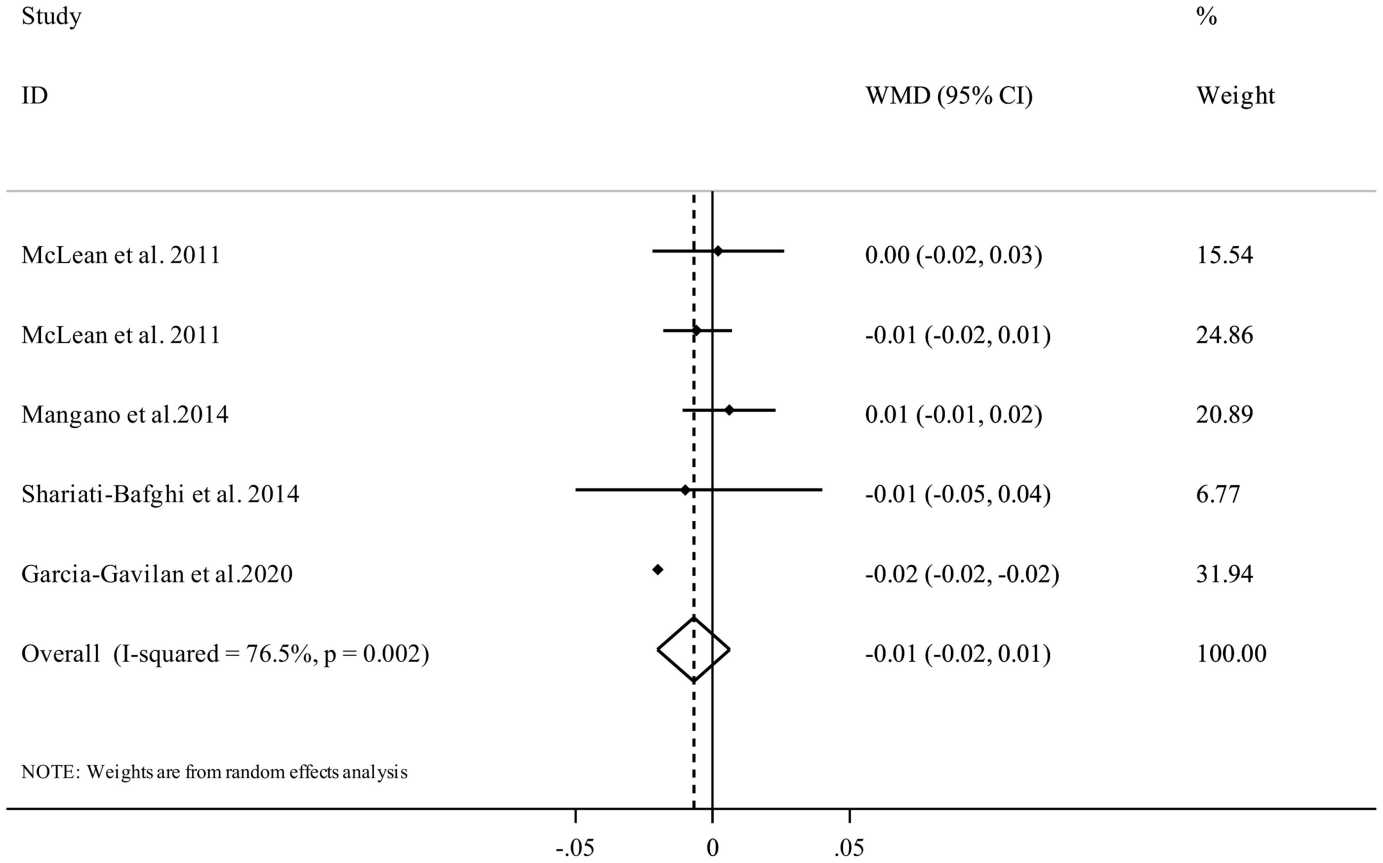
Forest plot for the association between PRAL and femoral BMD in adults aged >18 years expressed as weighted mean difference (WMD). Horizontal lines represent 95% CIs. Diamonds represent pooled estimates from random-effects analysis. BMD, Bone Mineral Density; CI, confidence interval.

Five studies from four publications (17, 21, 34, 35) investigated the association between PRAL and femoral BMD and included 7,216 participants. The random-effects meta-analysis revealed that there was no clear significant association between dietary PRAL score and BMD (WMD = −0.01, 95% confidence interval: −0.02 to 0.01, *I*^2^ = 76.5%, P_heterogeneity_ = 0.002) (Figure 4).

**Figure 5:**
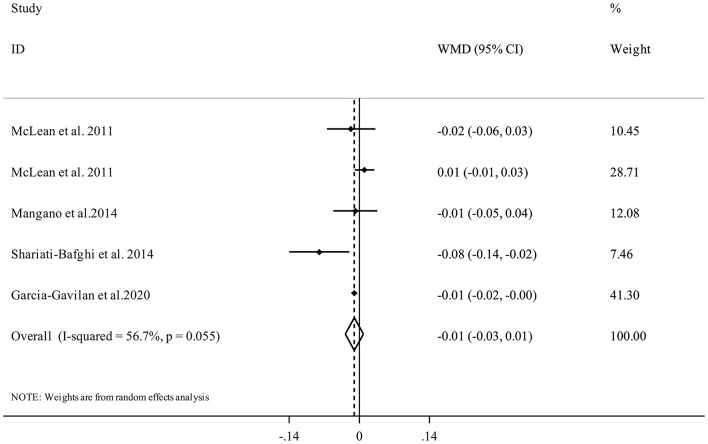
Forest plot for the association between PRAL and spinal BMD in adults aged >18 years expressed as weighted mean difference (WMD). Horizontal lines represent 95% CIs. Diamonds represent pooled estimates from random-effects analysis. BMD, Bone Mineral Density; CI, confidence interval.

Five studies (17, 21, 34, 35) with 6,309 participants examined the association between PRAL and spinal BMD. Performing meta-analysis indicated that higher dietary PRAL score was not associated with spinal BMD (WMD = −0.01, 95% CI: −0.03 to 0.01, *I*^2^ = 56.7%, P_heterogeneity_ = 0.05) (Figure 5).

#### NEAP and BMD

**Figure 6:**
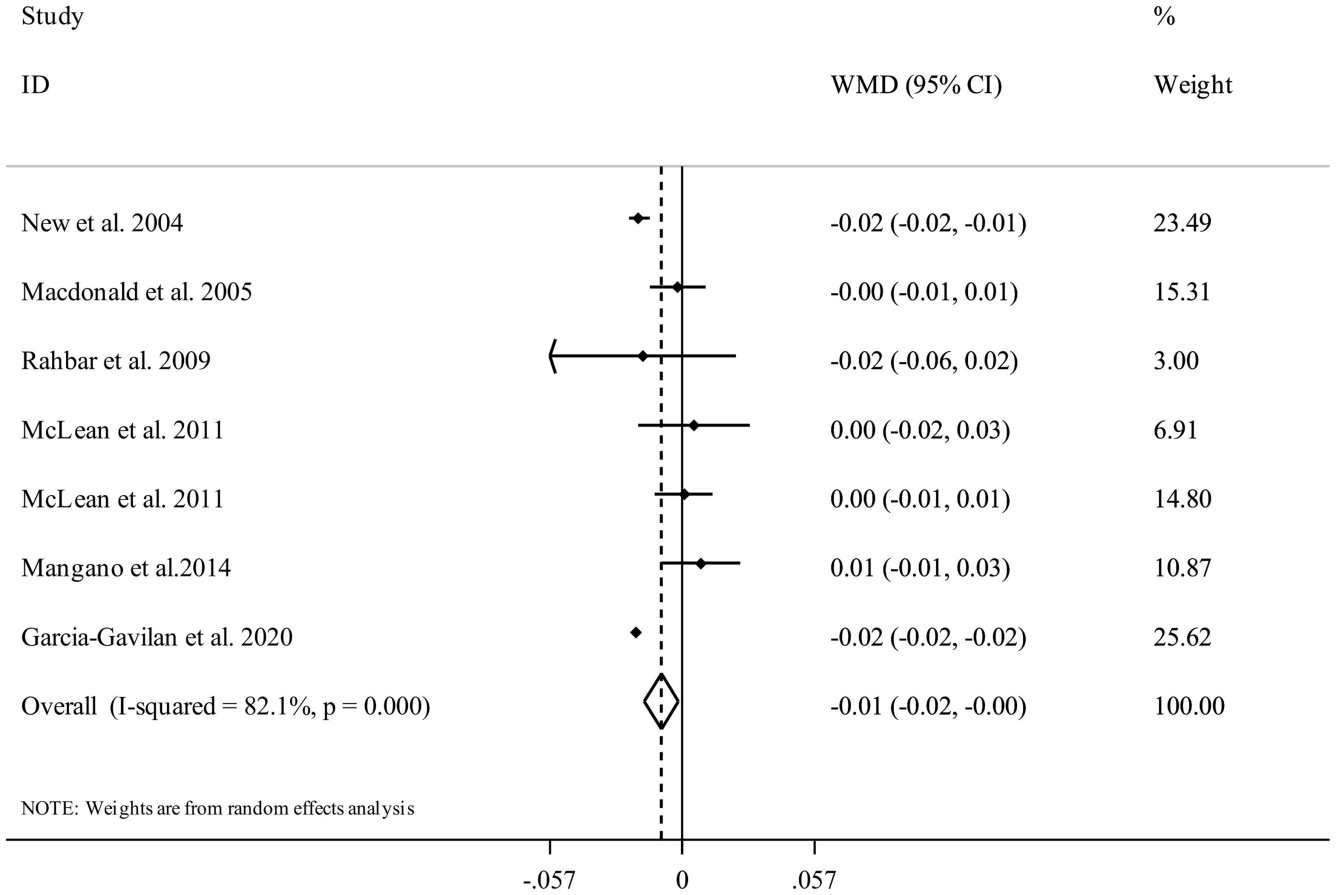
Forest plot for the association between NEAP and femoral BMD in adults aged >18 years expressed as weighted mean difference (WMD). Horizontal lines represent 95% CIs. Diamonds represent pooled estimates from random-effects analysis. BMD, Bone Mineral Density; CI, confidence interval.

Seven studies (17, 21, 29, 30, 33, 34) were included in the analysis of NEAP and femoral BMD. Summarizing data from 11,452 participants of these studies showed that having higher DAL content in terms of high NEAP scores was significantly associated with a lower femoral BMD (WMD = −0.01, 95% CI: −0.02 to −0.00, *I*^2^ = 82.1%, P_heterogeneity_ <0.001) (Figure 6).

**Figure 7:**
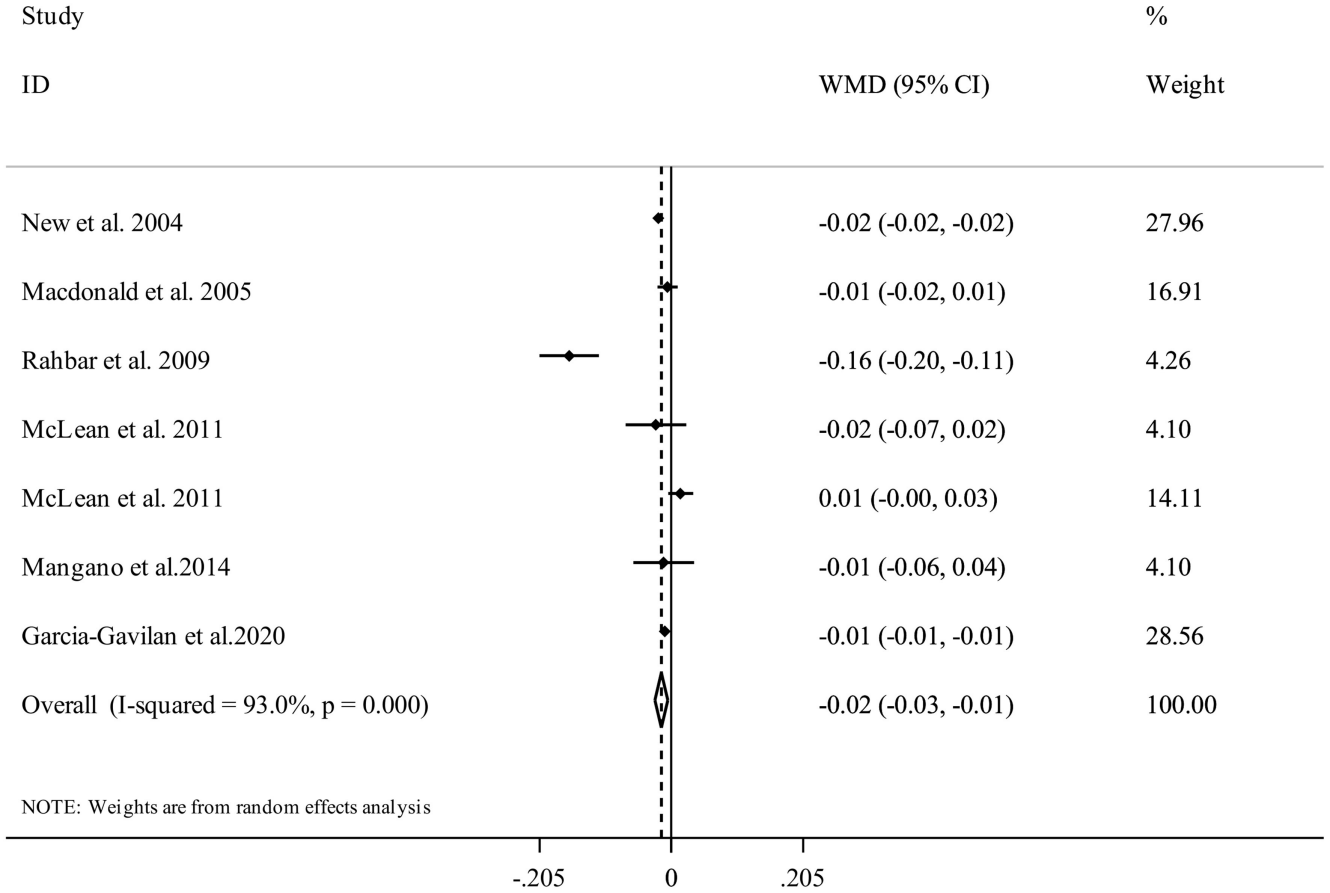
Forest plot for the association between NEAP and spinal BMD in adults aged >18 years expressed as weighted mean difference (WMD). Horizontal lines represent 95% CIs. Diamonds represent pooled estimates from random-effects analysis. BMD, Bone Mineral Density; CI, confidence interval.

Overall, there were seven studies from six publications with 10,308 participants (17, 21, 29, 30, 33, 34) for the relation between NEAP and spinal BMD. Combining data from these studies indicated that having higher DAL content in terms of high NEAP scores was significantly associated with a lower spinal BMD (WMD = −0.02, 95% CI: −0.03 to −0.01, *I*^2^ = 93%, P_heterogeneity_ <0.001) (Figure 7).

### Subgroup Analyses, Sensitivity Analyses, and Publication Bias

Supplementary Table 3 shows findings based on subgroup analyses of NEAP and BMD. The results demonstrated a significant inverse association between NEAP and Femoral neck BMD in studies that included both sex and females, conducted in non-US countries, used FFQ for dietary assessment, not adjusted for alcohol consumption, and presented weight-adjusted risk estimates. In terms of NEAP and Femoral neck BMD, a significant inverse association was seen in studies among females with a mean age of <60, conducted in non-US countries, used FFQ for dietary assessment, not adjusted for some potential confounders (energy and alcohol intake and smoking), and presented weight-adjusted risk estimates. Publication bias tests were not performed (<10 studies). Findings from the sensitivity analysis showed that excluding the studies by New et al. (29) and Garcia-Gavilan et al. (21) altered the significant inverse association between NEAP and spinal BMD to a non-significant association. Sensitivity analyses for the other associations showed that excluding any single study from the analysis did not appreciably alter the pooled effect sizes.

## Discussion

The present study is the first meta-analysis summarizing observational evidence on the association between indices of diet-dependent acid load and risk of fractures and BMD. Our results showed no significant association between DAL, represented by PRAL and NEAP, and risk of fractures. In addition, we found that high NEAP scores were significantly associated with a slightly lower femoral and spinal BMD. However, there was no significant association between dietary PRAL score and BMD.

The social and economic burden of osteoporotic fractures is increasing worldwide as the population ages. Fracture prevention has therefore become a major public health priority. Dietary variables have an important role in these conditions. Some diet components might have potential effects on the acid-base balance of the body due to the PH-altering features (38). Animal foods such as meat, fish, chicken, cheese, egg, and cereals are good sources of sulfur amino acids, phosphate, and chloride and are considered acid-forming foods. However, foods providing base precursors such as fruits and vegetables are rich in glutamate, citrate, and malate. Therefore, dietary patterns rich in animal foods and low in plant foods like Western-style dietary patterns might alter the body's acid-base balance (38). According to the regulation of acid-base balance in a narrow range, any imbalances in blood pH or bicarbonate buffer can affect overall health.

The current study provides reliable evidence on the association between DAL and bone health. We failed to find a clear association between DAL (based on PRAL and NEAP) and risk of fractures. A meta-analysis of cohort studies that examined the association of protein intake and bone health throughout the life course reported that total, animal, and plant protein intake was not associated with risk of osteoporotic fractures in healthy adults (39). A prospective cross-sectional analysis showed that urinary citrate, a biomarker for diet- and metabolism-dependent acid-base status, was inversely associated with fracture risk in adult females but not in males (40). In another meta-analysis, adopting “Healthy” dietary patterns reduced the risk of fractures, while the risk of fracture could be increased with the “Meat/Western” dietary pattern (41). Moreover, long-term observational studies documented that a high intake of some alkaline foods such as fruits and vegetables was associated with a lower risk of fracture (42). In the interpretation of these discrepancies, it must be considered that other dietary components and biologically active factors in the acid/base-forming foods and dietary patterns could also affect bone fracture. Further investigations are needed to find the exact relationship between DAL and risk of fractures.

The present review found that NEAP was inversely associated with a slightly lower BMD. However, there was no significant relationship between PRAL and spinal and femoral BMD. It appears that the different nutrients used in the calculation of NEAP and PRAL could be a contributing factor to the observed variations (43). Compared to NEAP, PRAL measures absorption rates of contributing nutrients from the gut (43). Moreover, the study by Remer et al. indicated that PRAL values were significantly correlated with net acid excretion in 24-h urine samples compared with NEAP (8). A systematic review and meta-analysis did not support the acid-ash hypothesis and concluded there is no evidence that an alkaline diet is a protective determinant of bone health (22). This hypothesis states that acid-forming foods like those found in the modern diet cause osteoporosis and alkaline-forming foods or supplements prevent osteoporosis. However, that study did not conduct any further statistical analysis but only applied the Hill standard review to retrospectively describe the relationship between acid load and bone metabolism. In an additional meta-analysis of 13 randomized clinical trials, alkaline supplementation had beneficial effects on bone metabolism and acidic diets were not harmful for bone health (44). Several randomized controlled trials have reported that high-protein diets which are considered acid-producing diets, have no significant effect on bone turnover markers and BMD (45, 46). Finding from an umbrella review indicated that an appropriate intake of dairy products, vegetables, fruits, and micronutrients and a lower intake of alcohol and coffee could reduce the risk of osteoporosis (47). It is possible that fruit and vegetables may be beneficial for bone health through mechanisms other than via the acid-base balance. Further studies should examine whether fruit and vegetables protect from the bone.

Several mechanisms have been proposed to explain how DAL might affect bone health (13, 48, 49). It is well known that bone acts as the primary buffering system for alkali components such as calcium and potassium in case of systemic acidosis (50). On the other hand, metabolic acidosis releases calcium from the bone matrix, weakening the bone and making it more prone to fracture by increasing osteoclastic resorption to maintain homeostasis (9, 10). Metabolic acidosis can also affect bone health by reducing insulin sensitivity and impairing glucose homeostasis, leading to inflammation and oxidative stress (51, 52). Thus, insulin resistance and inflammation are considered potential risk factors to explain the association between acidity and bone health.

### Strengths and Limitations

Our meta-analysis has several strengths, including a comprehensive literature search on the association between DAL and bone health. Another advantage is that we evaluated the associations between PARL and NEAP separately. However, several limitations should be acknowledged. While all included studies except four have high quality based on NOS, causality cannot be established due to the observational nature of the included studies. Therefore, this review for a definitive conclusion emphasizes adding high-quality intervention studies. Secondly, residual confounders due to unmeasured behavioral and biological factors or measurement errors in covariates cannot be completely ruled out. Thirdly, in most studies, food and nutrient intakes were assessed by food frequency questionnaires, measurement errors were inevitable. Fourth, we did not perform subgroup analysis for some outcomes because of the low number of related reports. Fifth, different methods for BMD assessment were used in the included studies, and the units of

BMD varied in different studies. Finally, there was some evidence of considerable heterogeneity among included studies which could be explained by variations in the amount of DAL, follow-up period, exposure assessment methods, and adjustments for confounding factors.

## Conclusion

In the current meta-analysis, adherence to diets with high acidity potential was not associated with risk of fractures. Furthermore, we found a significant weak association between NEAP and BMD. However, DAL based on PRAL was not associated with BMD. These findings thus have important public health implications. The current study provides further evidence that higher DAL is not associated with fracture risk and is weakly associated with BMD. However, these findings should be interpreted with caution and further large intervention trials are required.

## Data Availability Statement

The original contributions presented in the study are included in the article/Supplementary Materials, further inquiries can be directed to the corresponding author.

## Author Contributions

FG conceived and designed the study, conducted systematic search, screened and selected eligible articles, data interpretation, and manuscript drafting. SN performed analyses and interpreted results, data interpretation, and manuscript drafting. MS and NR screened and selected eligible articles and extracted information. KM is the guarantor. All authors contributed to writing, reviewing or revising the paper and have approved it for publication.

## Conflict of Interest

The authors declare that the research was conducted in the absence of any commercial or financial relationships that could be construed as a potential conflict of interest.

## Publisher's Note

All claims expressed in this article are solely those of the authors and do not necessarily represent those of their affiliated organizations, or those of the publisher, the editors and the reviewers. Any product that may be evaluated in this article, or claim that may be made by its manufacturer, is not guaranteed or endorsed by the publisher.
