# Supplementary material for: Dietary Acid Load and Bone Health: A Systematic Review and Meta-Analysis of Observational Studies
Source: Front Nutr. 2022 May 6;9:869132. doi: 10.3389/fnut.2022.869132 (PMC9120865; doi:10.3389/fnut.2022.869132)
Supplement: Supplementary file 1 [file Table_1.DOCX]

**Supplemental Table 1**: Medical subject headings (MeSH) and non-MeSH terms used to search relevant publications on the relation between DAL and bone health^1^

| **Concept 1** | (Bone OR "Fractures, Bone" OR fractures OR fracture OR BMD OR “bone mineral density” OR “bone turnover” OR Osteoporosis OR Osteopenia OR “Bone health” OR BMC OR “bone mineral content” OR “Bone Density” OR “bone mass density”) |
| --- | --- |
| **Concept 2** | (“Dietary acid load” OR “dietary acid-base load” OR “dietary acidity” OR “net acid load” OR “acid excretion” OR “potential renal acid load” OR PRAL OR “net endogenous acid production” OR NEAP OR “protein to potassium ratio” OR “protein/ potassium ratio” OR “potential renal acid load”) |

^1^The combination of terms as mentioned above was used to search online databases: ("concept 1" AND "concept 2")

**Supplemental Table 2:** Characteristics of observational studies included in the current systematic review and meta-analysis

| Study | Design | Country | Age | Sample size | DAL assessment  method | Dietary assessment tool | Outcome | BMD (means± SDs) | |  |  | NOS score |
| --- | --- | --- | --- | --- | --- | --- | --- | --- | --- | --- | --- | --- |
|  |  |  |  |  |  |  |  | Lowest category | Highest category | RR (95 % CI) | B |  |
| McLean et al. 2011 | Cross-sectional (Framingham Original study) | US | 68-92 | M:371 | PRAL | FFQ | Femoral neck BMD  Lumbar spine BMD | 0.889±0.173  1.292±0.336 | 0.859±0.166  1.295±0.332 | - | - | 8 |
|  |  |  |  | F:587 |  |  | Femoral neck BMD  Lumbar spine BMD | 0.716±0.122  1.110±0.244 | 0.728±0.120  1.087±0.239 |  |  |  |
|  |  |  |  | M:371 | NEAP |  | Femoral neck BMD  Lumbar spine BMD | 0.885±0.174  1.276±0.338 | 0.842±0.159  1.251±0.328 |  |  |  |
|  |  |  |  | F:587 |  |  | Femoral neck BMD  Lumbar spine BMD | 0.716±0.122  1.103±0.231 | 0.737±0.121  1.079±0.242 |  |  |  |
|  | Cross-sectional (Framingham Offspring Study) |  | 35-86 | M:1269 | PRAL |  | Femoral neck BMD  Lumbar spine BMD | 0.970±0.125  1.327±0.196 | 0.970±0.125  1.304±0.196 | - | - | 8 |
|  |  |  |  | F:1611 |  |  | Femoral neck BMD  Lumbar spine BMD | 0.865±0.120  1.157±0.180 | 0.875±0.120  1.159±0.180 |  |  |  |
|  |  |  |  | M:1269 | NEAP |  | Femoral neck BMD  Lumbar spine BMD | 0.972±0.124  1.315±0.213 | 0.975±0.125  1.338±0.196 |  |  |  |
|  |  |  |  | F:1611 |  |  | Femoral neck BMD  Lumbar spine BMD | 0.867±0.120  1.157±0.181 | 0.866±0.121  1.148±0.180 |  |  |  |
| Garcia-Gavilan et al.2020 | Cross-sectional  (PREDIMED-Plus) | Spain | 55-80 | M/F: 1102 | PRAL | FFQ | Total Femur BMD  Femoral neck BMD  Lumbar spine BMD  Trochanter BMD  Femoral diaphysis | 1.100±0.007  0.930±0.007  1.170±0.010  0.840±0.006  1.120±0.009 | 1.030±0.007  0.910±0.007  1.160±0.010  0.870±0.006  1.240±0.008 | - | - | 8 |
|  |  |  |  |  | NEAP |  | Total Femur BMD  Femoral neck BMD  Lumbar spine BMD  Trochanter BMD  Femoral diaphysis | 1.010±0.007  0.930±0.007  1.170±0.010  0.840±0.006  1.220±0.008 | 1.030±0.007  0.910±0.007  1.160±0.010  0.870±0.006  1.240±0.008 | - | - |  |
|  | Longitudinal study (PREDIMED) |  |  | M/F: 870 | PRAL |  | Fracture | - | - | 1.91 (1.14-3.19) | - | 6 |
|  |  |  |  |  | NEAP |  |  |  |  | 1.87 (1.1-3.17) |  |  |
| Mangano et al.2014 | Cohort (NHANES) | US | ≥60 | M: 1218 | PRAL | Food recall | Femoral neck BMD  Lumbar spine BMD | 0.780±0.134  1.084±0.302 | 0.776±0.167  1.076±0.260 | - | - | 8 |
|  |  |  |  | F: 907 |  |  | Femoral neck BMD  Proximal Femur BMD | 0.706±0.113  0.847±0.154 | 0.723±0.154  0.842±0.113 |  |  |  |
|  |  |  |  | M: 1218 | NEAP |  | Femoral neck BMD  Lumbar spine BMD | 0.780±0.134  1.072±0.306 | 0.783±0.153  1.084±0.302 | - | - |  |
|  |  |  |  | F: 907 |  |  | Femoral neck BMD  Proximal femur BMD | 0.706±0.119  0.849±0.159 | 0.722±0.175  0.836±0.119 |  |  |  |
| Macdonald et al. 2005 | Cross-sectional | Scotland | 54.9 | F: 2929 | NEAP | FFQ | Femoral neck BMD  Lumbar spine BMD | 0.833±0.120  1.002±0.160 | 0.835±0.120  1.008±0.170 | - | - | 5 |
| New et al. 2004 | Cross-sectional | UK | 45-54 | F: 1056 | NEAP | FFQ | Femoral neck BMD  Lumbar spine BMD  Forearm total BMD  Femoral Ward’s area BMD | 0.893±0.029  1.054±0.016  0.382±0.014  0.824±0.039 | 0.874±0.027  1.074±0.016  0.417±0.014  0.852±0.047 | - | - | 6 |
| Rahbar et al. 2009 | Cross-sectional | Iran | 20-72 | F: 165 | NEAP | FFQ | Femoral neck BMD  Lumbar spine BMD  Distal third of radius BMD | 0.947±0.142  0.739±0.164  0.331±0.108 | 0.964±0.149  0.898±0.165  0.501±0.110 | - | - | 5 |
| Hayhoe et al. 2020 | Cohort (EPIC-Norfolk) | UK | 59.7 | M: 11511 | PRAL | Food diary | BUA  Fracture | 90.02±17.33 | 90.37±17.29 | 1.33 (1.03-1.72) | - | 7 |
|  |  |  | 58.9 | F: 13927 |  |  | BUA  Fracture | 72.78±14.11 | 71.67±14.07 | 1.20 (1.02-1.41) |  |  |
| Dargent-Molina et l. 2008 | Cohort (E3N) | France | 56.1 | F: 36217 | PRAL | Food recall | Fracture | - | - | 1.05 (0.93-1.19) |  | 7 |
| Jia et al. 2014 | Cohort (PIVUS) | Sweden | 70 | M/F: 861 | PRAL | Food record | Total body T-score  Lumbar spine T-score  Femoral neck T-score  Total hip T-score  Fracture | - | - | 0.93 (0.55-1.55) | -0.02  -0.02  -0.02  -0.02 | 6 |
|  |  |  |  |  | NEAP |  | Total body T-score  Lumbar spine T-score  Femoral neck T-score  Total hip T-score  Fracture | - | - | 1.03 (0.57-1.85) | -0.05  -0.03  -0.05  -0.06 |  |
| Papageorgiou et al. 2020 | Cross-sectional | Denmark | 65.1 | M/F: 704 | PRAL | Food record | Lumbar spine BMD  Total hip BMD  Total radius BMD | - | - | - | 0.0008  0.0001  -0.0001 | 7 |
| De Jonge et al.2017 | Cross-sectional | Netherlands | 52-82 | M/F: 4672 | NEAP | FFQ | Trabecular bone score  Bone mineral density | - | - | - | -0.04  -0.02 | 8 |
| Wynn et al. 2008 | Cross-sectional | Swiss | 80.6 | F: 256 | NEAP | FFQ | BUA | 98.8±9.5 | 95.6±1.1 | - | - | 4 |
| Thorpe et al. 2008 | Cross-sectional | US | 67.9 | F: 161 | PRAL | Food recall | Lumbar spine BMD  Total hip BMD | - | - | - | -0.03  0.12 | 4 |
|  |  |  |  |  | NEAP |  | Lumbar spine BMD  Total hip BMD |  |  |  | -0.03  0.08 |  |
| Shariati-Bafghi et al. 2014 | Cross-sectional | Iran | 50-85 | F: 151 | RNAE | FFQ | Femoral neck BMD  Lumbar spine BMD | 0.67+0.09  0.90+0.12 | 0.66+0.09  0.82+0.14 | - | - | 5 |

Abbreviation: DAL: dietary acid load- BMD: bone mineral density- NEAP: net endogenous acid production- PRAL: potential renal acid load- BUA: broadband ultrasound attenuation- CI: confidence interval- M: male- F: female- FFQ: food frequency questionnaire- US: United States- RR: risk ratio

**Supplemental Table 3:** Subgroup analysis for the association of NEAP and BMD

|  |  | Femoral neck BMD | | | | Lumbar spine BMD | | | |
| --- | --- | --- | --- | --- | --- | --- | --- | --- | --- |
|  |  | ES (95% CI) | N^2^ | I^2^ (%) | P-heterogeneity^4^ | ES (95% CI)^3^ | ES, n | I^2^ (%) | P-heterogeneity |
| Gender | |  |  |  |  |  |  |  |  |
|  | Both | -0.003 (-0.020 to -0.014) | 4 | 88.1 | <0.001 | -0.005 (-0.020 to 0.011) | 4 | 51.3 | 0.10 |
|  | female | -0.012 (-0.026 to -0.002) | 3 | 70.4 | 0.03 | -0.051 (-0.091 to -0.010) | 3 | 94.7 | <0.001 |
| Study location | |  |  |  |  |  |  |  |  |
|  | US | 0.004 (-0.006 to 0.013) | 3 | 0 | 0.80 | 0.001 (-0.022 to 0.025) | 3 | 27.9 | 0.25 |
|  | Non-US | -0.017 (-0.022 to -0.012) | 4 | 65.7 | 0.03 | -0.021 (-0.032 to -0.009) | 4 | 96.2 | <0.001 |
| Age | |  |  |  |  |  |  |  |  |
|  | Age≥60 | -0.003 (-0.020 to 0.014) | 4 | 88.1 | <0.001 | -0.005 (-0.020 to 0.011) | 4 | 51.3 | 0.10 |
|  | Age<60 | -0.012 (-0.026 to 0.002) | 3 | 70.4 | 0.03 | -0.051 (-0.091 to -0.010) | 3 | 94.7 | <0.001 |
| Dietary assessment tools | |  |  |  |  |  |  |  |  |
|  | FFQ | -0.012 (-0.019 to -0.005) | 6 | 78.6 | <0.001 | -0.016 (-0.027 to -0.005) | 6 | 94.1 | <0.001 |
|  | Food recall | 0.008 (-0.009 to 0.025) | 1 | - | - | -0.012 (-0.059 to 0.035) | 1 | - | - |
| Adjustment for physical activity | |  |  |  |  |  |  |  |  |
|  | Yes | -0.003 (-0.020 to 0.014) | 4 | 88.1 | <0.001 | -0.005 (-0.020 to 0.011) | 4 | 51.3 | 0.10 |
|  | No | -0.012 (-0.026 to 0.002) | 3 | 70.4 | 0.03 | -0.051 (-0.091 to -0.010) | 3 | 94.7 | <0.001 |
| Adjustment for smoking | |  |  |  |  |  |  |  |  |
|  | Yes | -0.003 (-0.020 to 0.014) | 4 | 88.1 | <0.001 | -0.005 (-0.020 to 0.011) | 4 | 51.3 | 0.10 |
|  | No | -0.012 (-0.026 to 0.002) | 3 | 70.4 | 0.03 | -0.051 (-0.091 to -0.010) | 3 | 94.7 | <0.001 |
| Adjustment for alcohol | |  |  |  |  |  |  |  |  |
|  | Yes | 0.004 (-0.006 to 0.013) | 3 | 0 | 0.80 | 0.001 (-0.022 to 0.025) | 3 | 27.9 | 0.25 |
|  | No | -0.017 (-0.022 to -0.012) | 4 | 65.7 | 0.03 | -0.021 (-0.032 to -0.009) | 4 | 96.2 | <0.001 |
| Adjustment for energy | |  |  |  |  |  |  |  |  |
|  | Yes | -0.003 (-0.020 to 0.014) | 4 | 88.1 | <0.001 | -0.005 (-0.020 to 0.011) | 4 | 51.3 | 0.10 |
|  | No | -0.012 (-0.026 to 0.002) | 3 | 70.4 | 0.03 | -0.051 (-0.091 to -0.010) | 3 | 94.7 | <0.001 |
|  | Adjustment for weight |  |  |  |  |  |  |  |  |
|  | Yes | -0.011 (-0.018 to -0.003) | 6 | 80.2 | <0.001 | -0.018 (-0.029 to -0.006) | 6 | 94.1 | <0.001 |
|  | No | -0.002 (-0.014 to 0.010) | 1 | - | - | -0.006 (-0.022 to 0.010) | 1 | - | - |

^1^Abbreviation: BMI, body mass index; CI, confidence interval; FFQ, food frequency questionnaire; US, United States

^2^Number of risk estimates

^3^Obtained from the random-effects model

^4^Inconsistency- the percentage of variation across studies due to heterogeneity

^5^Obtained from the Q-test
